# Diagnostic Challenges in the Neuropsychology of Epilepsy: Report of the ILAE Neuropsychology Task Force Diagnostic Methods Commission: 2021–2025

**DOI:** 10.1002/epd2.70052

**Published:** 2025-06-13

**Authors:** Mary Lou Smith, Mayu Fujikawa, Genevieve Rayner, Seth A. Margolis, Bruce Hermann, Gus Baker, Sallie Baxendale, Robyn Busch, Aimee Dollman, Vicki Ives‐Deliperi, Carrie R. McDonald, Urvashi Shah, Sarah Wilson

**Affiliations:** ^1^ Department of Psychology University of Toronto; Neurosciences and Mental Health Program, the Hospital for Sick Children Toronto Ontario Canada; ^2^ Neurosciences and Mental Health Program, the Hospital for Sick Children Toronto Ontario Canada; ^3^ Department of Epileptology Tohoku University Graduate School of Medicine Sendai Japan; ^4^ Melbourne School of Psychological Sciences The University of Melbourne Melbourne Victoria Australia; ^5^ Comprehensive Epilepsy Program The Alfred Hospital Melbourne Victoria Australia; ^6^ Department of Psychiatry Brown University Health Providence Rhode Island USA; ^7^ Department of Psychiatry and Human Behavior, Warren Alpert Medical School of Brown University Providence Rhode Island USA; ^8^ Department of Neurology University of Wisconsin School of Medicine and Public Health Madison Wisconsin USA; ^9^ Division of Neurosciences University of Liverpool Liverpool UK; ^10^ Department of Clinical and Experimental Epilepsy Institute of Neurology, UCL London UK; ^11^ Epilepsy Center and Department of Neurology, Neurological Institute Cleveland Clinic Cleveland Ohio USA; ^12^ Neuroscience Institute University of Cape Town Cape Town South Africa; ^13^ Division of Psychiatry, Neuroscience Institute University of Cape Town Cape Town South Africa; ^14^ Center for Multimodal Imaging and Genetics UC San Diego San Diego California USA; ^15^ Department of Neurology King Edward Memorial Hospital Mumbai India; ^16^ Victorian Collaborative Centre for Mental Health and Wellbeing Melbourne Victoria Australia

**Keywords:** comorbidity, neuropsychology, psychiatric, psychosocial

## Abstract

Increasingly, it has been recognized that non‐seizure‐related factors influence how people with epilepsy perform on neuropsychological tests. Therefore, neuropsychologists need to recognize the constellation of factors that can contribute to the neurocognitive presentation of a person with epilepsy and consider these factors in the interpretation of their assessment results. In this paper, we highlight common scenarios prompting the need to account for such factors when conducting and interpreting neuropsychological assessments. To illustrate these complexities, a case study is presented of a woman with early onset epilepsy complicated by a traumatic brain injury in adulthood who was a candidate for epilepsy surgery. A discussion of the need to consider neurodevelopmental, psychiatric, and psychosocial factors in conducting an assessment and arriving at a sound interpretation of results is presented. Clinical take‐away points are offered for guidance in undertaking such assessments.

**Key points**

- Neuropsychologists in the field of epilepsy need expert knowledge to shape their ability to make appropriate diagnoses and recommendations.
- Multiple non‐seizure‐related factors contribute to performance on neuropsychological tests.
- Clinicians must recognize and incorporate these factors into their judgements and diagnostic formulations.

## 1 INTRODUCTION

Early in the development of neuropsychology as a discipline, emphases within epilepsy focused on two critical approaches. One approach was on mapping deficits onto models of localized and lateralized functional representation within the brain. The other approach emphasized understanding cognitive dysfunction in relation to the taxonomy of epilepsy, in which disease characteristics (e.g., etiology, age at seizure onset, duration of epilepsy, anti‐seizure medications) were investigated as predictors of impairments. Although both approaches yielded considerable information about the nature of cognitive and neurobehavioral deficits in people with epilepsy, advances in neuropsychological research revealed limitations to these types of investigations. It became apparent that there are similar cognitive impairments seen across epilepsy syndromes, reflecting often widespread distributed structural abnormalities in gray and white matter, and consistent with the more recent conceptualization of epilepsy as a network disorder.^1^ In addition, there is now a considerable body of literature on how non‐seizure‐related factors influence performance on neuropsychological tests (e.g., socioeconomic status, psychiatric comorbidities, psychosocial influences). For these reasons, neuropsychologists need to recognize the myriad of factors that can contribute to the neurocognitive presentation of a person with epilepsy and to consider these factors in the interpretation of their assessment results. Many of these same factors can also influence the test performance of individuals with other neurological disorders (Appendix S2).

This paper builds on previous work by members of the International League Against Epilepsy Diagnostic Commission Neuropsychology Task Force that provided an overview of indications and expectations for neuropsychological assessment in routine epilepsy care and in epilepsy surgery.^2^, ^3^ This paper addresses the complex interplay of neurodevelopmental, psychiatric, and psychosocial factors that can influence performance during a neuropsychological assessment and the resulting patterns of test scores. These factors frequently result in diagnostic challenges that need to be acknowledged and at times set limits on the conclusions that can be drawn from the assessment. The paper is not intended to provide an exhaustive review of the current state of neuropsychological considerations or outcomes in epilepsy but rather to highlight common scenarios prompting the need to account for such factors when conducting and interpreting neuropsychological assessments. The content set out here presupposes and assumes a high level of familiarity on the part of the reader of neuropsychological assessment practices, methods, tests, their purpose, and their interpretation.

We begin with the case of a surgical patient for whom many such considerations were relevant. Thereafter, we provide information across three domains (neurodevelopmental factors, psychiatric factors, and psychosocial factors) to reflect on the potential challenges associated with the approach to assessment and interpretation in this case. We underscore how one may work to tease apart the complexity of such diagnostic challenges and reason through them to address patients' needs in clinical practice. Clinical “take‐aways” are offered following each of these sections to help guide clinicians in their own practice.

## 2 CASE PRESENTATION

The following case illustration was selected to highlight how various individual and clinical challenges can influence the neuropsychological assessment, diagnosis, prognosis, and care plans of people with epilepsy.

Ms. X is a bilingual (primarily Spanish speaking) woman with medication‐resistant epilepsy. She was seen for a neuropsychological assessment at the age of 43 years in advance of epilepsy surgery for presumed right temporal lobe epilepsy. Table 1 summarizes the details of her seizure history and MRI.

**TABLE 1:** Details of Ms. X's seizure history.

Age of onset	Infancy (with febrile seizures)
Seizure type	Focal seizures with impaired awareness
Seizure history	Seizure free from ages 31–41 years on three anti‐seizure medications—levetiracetam: 1000 mg tablets, two tablets twice daily (total daily dose: 4000 mg); clonazepam: 1 mg, once daily; topiramate: 200 mg tablets, twice daily (total daily dose: 400. Seizures recurred with onset of early menopause and during a time of heightened stress associated with the death of her grandmother (who helped raise her).
Pre‐surgical EEG	Routine EEG showed sharp waves in the right temporal lobe and intermittent focal slowing in the right frontotemporal region, suggestive of an epileptogenic focus in the right temporal region.
Pre‐surgical MRI	Findings consistent with right mesial temporal sclerosis (see Figures S1 and S2 for MRI images)

Numerous clinical intricacies added diagnostic challenges to Ms. X's neuropsychological workup and prognosis. At the age of 34, she suffered a severe traumatic brain injury (TBI). She was a fastened passenger in a car‐on‐tree collision. She was unresponsive at the scene (Glascow Coma Scale (GCS) = 7), intubated, and admitted to the trauma ICU where she began following commands intermittently 3 days later. After 2 weeks, she was transferred to a step‐down unit with a GCS of 10, indicating a moderate brain injury with responses limited to eye opening and motor movements. She then underwent acute inpatient rehabilitation, which included occupational and physical therapy to address balance, cognitive, and safety concerns. Ultimately, she was discharged home with 24‐hour supervision from family and in‐home rehabilitation services. She does not recall her accident or the hospital stay.

Neuroimaging from that time revealed intraventricular subarachnoid hemorrhage (left > right), subcortical left frontal intraparenchymal contusions, and a parenchymal contusion within the posterior left temporal lobe. Though Ms. X was *left*‐handed from birth (no family history of left‐handedness), orthopedic injuries sustained during her MVA forced her to become right‐handed. Post‐acute brain MRI, obtained 3 years later, revealed focal hemosiderin deposition within the *left* hippocampus and a contemporaneous routine EEG revealed intermittent slowing in the left temporal region *without* epileptiform discharges or electrographic seizures. However, another routine EEG obtained in the context of her pre‐surgical workup showed sharp waves in the right temporal lobe and intermittent focal slowing in the right frontotemporal region, suggestive of an epileptogenic focus in the right temporal region.

Ms. X's seizure semiology did not change following her TBI, but her TBI negatively impacted her functioning. She had graduated high school in South America, moved to the United States at age 21, and learned English through work and night school. She eventually entered community college to earn an associate's degree, where her classes were in English, and she completed 2.5 years of part‐time study before the TBI prevented her from returning for cognitive reasons. At the time of her pre‐surgical workup, she was working full time as an assembler in a package factory. Except for not driving due to seizures, she was functionally independent. She was motivated for epilepsy surgery because seizures imposed a negative effect on her social life, and she felt socially isolated and stigmatized. She reported that the return of her seizures “dramatically changed [her] social life.” Although she had friends at work, she lived alone, her family lived abroad, she was single and never married, and she did not have children. She hoped that epilepsy surgery could reduce her seizures and allow her to resume driving. She described her mood as “not too good,” but denied suicidal ideation, intent, plans, or attempts. She never abused substances.

Ms. X preferred that neuropsychological testing be conducted in Spanish. As there were no local Spanish‐speaking neuropsychologists with expertise in epilepsy in her area, she was evaluated at her local Level IV Epilepsy Center by a board‐certified clinical neuropsychologist with the assistance of a Spanish‐speaking medical interpreter and a Spanish‐speaking psychometrist. Test selection emphasized measures that would adequately sample verbal and visual memory while also disentangling the effects of her left hemisphere brain injuries. The test battery included measures from RBANS, NP‐NUMBRS,^4^ and the Color Trails Test^5^ because their normative samples offered age‐appropriate comparisons for a U.S. acculturated, primarily Spanish‐speaking late bilingual, with approximately 14 years of education.

Table 2 presents the scores obtained by Ms. X on all tests. Consistent with her frontal‐subcortical brain injuries, she demonstrated marked executive dysfunction with variable working memory and processing speed. Although her visual confrontation naming was intact, her verbal fluency was diminished, and it was unclear if this reflected left frontotemporal dysfunction versus an iatrogenic effect of topiramate.^6^ In line with her right MTS and presumed right temporal seizure focus, visual learning and memory were markedly impaired. However, her verbal learning and memory were also markedly deficient. In the context of her left hippocampal contusion/bleed, her neuropsychological test results raised concern about the functional reserve of her left hippocampus. A Spanish‐language Wada test with methohexital was subsequently performed and demonstrated left hemisphere language dominance and no evidence of right hemisphere language representation. Following right hemisphere inactivation, Ms. X correctly recognized 90% of previously shown memory stimuli. With left hemisphere inactivation, she recognized only 40% of previously shown memory stimuli. Considering the observed 50% memory asymmetry, it was inferred that Ms. X's left hemisphere would be able to support memory following a destructive right/nondominant temporal surgery involving the hippocampus. She underwent a right amygdalohippocampectomy via laser interstitial thermal therapy (see Figure S2 for post‐surgical MRI).

**TABLE 2:** Pre‐ and post‐surgical neuropsychological test performance.

Test	Pre‐surgical assessment		Post‐surgical assessment		Pre‐ to post‐comparison
	Raw score	Normed score	Raw score	Normed score	
Orientation					
Temporal	0 errors		0 errors		Stable
Spatial	0 errors		0 errors		Stable
Attention/Processing speed					
WAIS‐R arithmetic	5	*T* = 29	6	*T* = 33	Stable
PASAT	22	*T* = 41	21	*T* = 37	Stable
WAIS‐III					
Letter number sequencing	6	*T* = 33	5	*T* = 30	Stable
Digit symbol	69	*T* = 42	57	*T* = 38	Stable
Symbol search	26	*T* = 39	6	*T* = 17	Decline
Color Trails Test I (errors)	82 (0)	*z* = −1.7	122 (0)	*z* = −3.9	Decline
Executive					
Color trails 2	D/c (>240 s)		179 (0)	z = −1.4	Improved
Letter fluency^a^	23	*T* = 30	33	*T* = 41	Improved
WCST‐64					
Categories completed	0	—	1	—	Improved
Total errors	49	*T* = 19	36	*T* = 35	Improved
Perseverative errors	34	*T* = 26	22	*T* = 34	Stable
Conceptual level responses	3	*T* = 27	19	*T* = 39	Improved
Language					
RBANS picture naming	10/10	51–72 percentile	8/10	3–9 percentile	Decline
Animal fluency	12	*T* = 23	11	*T* = 20	Stable
Visuospatial					
RBANS figure copy	18	ss = 10	19	ss = 11	Stable
WAIS‐R block design	22	*T* = 46	27	*T* = 50	Stable
Memory					
Hopkins Verbal Learning Test					
Total learning trials 1–3	19 (5,6,8)	*T* = 31	21 (7,7,7)	*T* = 35	Stable
Delayed recall	1	*T* = 17	5	*T* = 28	Improved
Retention	12.5%	<1 percentile	71.4%	16–25 percentile	Improved
Recognition discrimination	8 (9,1)	9 percentile	10 (11,1)	25 percentile	Improved
RBANS					
Story memory	14 (8,6)	ss = 6	15 (6,9)	ss = 8	Stable
Story recall	0	ss = 1	5	ss = 4	Improved
Figure recall	0	ss = 1	0	ss = 1	Stable
Brief Visuospatial Memory Test					
Total learning trials 1–3	9 (2,3,4)	*T* = 26	12 (5,3,4)	*T* = 31	Stable
Delayed recall	3	*T* = 25	1	*T* = 18	Decline
Recognition discrimination	4 (4,0)	9 percentile	1 (1,0)	<1 percentile	Decline
Mood					
Beck depression inventory‐II	13		1		Improved

*Note*: Post‐surgical normed scores exhibiting a ±1 standard deviation difference compared to pre‐surgical levels were considered significantly different from baseline (i.e., improvement or decline) while those scores falling within 1 standard deviation were considered stable.

Abbreviations: PASAT, Paced Auditory Serial Attention Task; RBANS, Repeatable Battery for the Assessment of Neuropsychological Status; WAIS, Wechsler Adult Intelligence Scale; WCST, Wisconsin Card Sorting Test.

^a^ Letters used: P, M, R.

Ms. X enjoyed several months of seizure freedom, but eventually milder seizures of shorter duration recurred about once per week without auras or lengthy postictal symptoms. She reported a mild reduction in memory but did not experience further social, functional, or vocational disruptions. She had resumed driving to close by locations (going shopping, to do laundry).

Ms. X was seen six‐month post‐surgery for re‐evaluation (total test–retest interval was 829 days (approximately 2.27 years)). As seen in the post‐surgical columns of Table 2, Ms. X's visuomotor processing speed, visual confrontation naming, visual memory recall, and visual recognition memory declined. However, interpreting the degree of decline is challenging due to floor effects observed in her pre‐surgical testing (e.g., Figure Recall). She showed an improvement in executive functioning and verbal memory, suggesting a release of function to contralateral (left) and frontal‐subcortical abilities with a reduction in her seizure activity. Additionally, depression symptoms, which were mildly elevated pre‐surgically, dramatically improved.

## 3 NEURODEVELOPMENTAL ISSUES

### 3.1 Nature of the underlying neuropathology

Cognitive functions typically develop in a predetermined pattern across the lifespan. “Windows of opportunity” are periods in neurodevelopment when the brain is primed to develop specific functions. Different functions are prioritized at different stages of neurodevelopment when these windows of opportunity open and close. Once fully developed, cognitive functions also peak, plateau and decline at different times and at varying rates across the adult life span. For example, while the skills that comprise verbal comprehension typically continue to improve into the fifth decade of life before they begin to plateau, processing speed will have peaked and begun to decline decades earlier.^7^ Standardized neuropsychological tests take into account the developmental trajectories of cognitive function across the lifespan and essentially provide a measure of how someone functions in a specific cognitive domain compared to their age‐matched peers. When cognitive functions develop and decline normally, an individual's position in relation to their peers will remain stable across the lifespan. A neuropsychological assessment will establish where an individual functions in relation to their healthy, age‐matched peers (in terms of a percentile rank) across a range of cognitive domains. The profile will also highlight areas of focal impairment within an individual's profile.

In people with epilepsy, the nature of any underlying epileptogenic pathology plays a critical role in shaping the nature and extent of any associated neuropsychological deficit, often well before an individual has their first seizure. In the case of Ms. X, even though she was left‐handed from birth without a family history of left‐handedness, and bilinguals with left temporal lobe epilepsy have an increased risk of atypical language development and verbal memory,^8^ the likelihood that the right hemisphere contained eloquent language and verbal memory is still relatively low; indeed, the Wada test confirmed left hemisphere language representation. Contrast this with a hypothetical patient who has had a left temporal focal cortical dysplasia (FCD), a neurodevelopmental abnormality that is present from birth. The cortical representation of cognitive function would be different to that seen in Ms. X whose brain had presumably developed normally without this pathology. Neuroplasticity and reorganization of functions away from the abnormality may have resulted in a relatively mild impairment of functions traditionally associated with left temporal lobe function in the person with FCD compared to a brain that developed normally.

Consider another hypothetical patient who developed seizures following encephalitis at the age of 18 and had left hippocampal volume loss evident on MRI. This patient's brain developed normally until the onset of their illness, which coincided with their seizure onset. Their left hippocampus will have played a critical role in verbal memory functions prior to the onset of their illness and seizures. The damage to the left hippocampus associated with the encephalitis would have resulted in a marked impairment of verbal memory function because memory functions that developed normally were destroyed by the illness.

Each of these cases has temporal lobe epilepsy with structural abnormalities evident in the hippocampus. However, the developmental context interacts with the side of pathology to shape the nature and extent of the associated neuropsychological deficits in each case. This neurodevelopmental context does not just have implications for the interpretation of the preoperative neuropsychological profile. It is also critical in the prediction of postoperative change. A standardized resection can result in a wide variety of outcomes, depending on the functional status of the tissue removed and that remaining in situ.

### 3.2 Age of onset in childhood

Ms. X had the onset of her seizures as a young child. In her case, consideration of the developmental context is critical for several reasons. Earlier age at seizure onset is often associated with an increased risk for neuropsychological impairment, due to the neurodevelopmental context mentioned above, with the extent of the developmental lesion having an impact on the type and degree of neuroplasticity. Early onset epilepsy may be of genetic origin in many cases,^9^ a cause which itself can alter brain development and organization throughout the lifespan and leading to reorganization reducing certainty about how key cognitive and behavioral functions are mapped onto brain structures. Widespread patterns of neuropsychological impairment are commonly seen in childhood‐onset epilepsies and do not differentiate between different epilepsy syndromes.^10^ Thus, in addition to considering the neurodevelopmental context of the underlying pathology, neuropsychologists must also be cognizant of the impact of the age of seizure onset on development and function. Individuals with neurodevelopmental abnormalities may develop seizures at any stage in life. Seizure onset and commencement of anti‐seizure medications in infancy will impact broad aspects of social, cognitive, and educational development in a way that the onset of seizures in adulthood will not. Neuropsychologists must be aware of the neurodevelopmental context of both the underlying pathology and seizure onset when interpreting cognitive profiles.

Furthermore, pediatric‐onset epilepsy is frequently accompanied by other neurodevelopmental disorders which carry their own associations with cognitive and behavioral disorders. Rates of attention deficit hyperactivity disorder (ADHD) are 2.5–5.5 times higher than in healthy children without seizures.^11^ Despite this high rate, ADHD is often not diagnosed in childhood, and the disorder can persist into adulthood. The inattention in ADHD can have a negative impact on the development of other cognitive skills and the ability to benefit from structured learning opportunities. In individuals with epilepsy, academic and vocational underachievement, as well as depression and anxiety, can be associated with a history of ADHD. Autism spectrum disorder (ASD) similarly has a higher prevalence in individuals with epilepsy, and a recent review found that of possible factors associated with the occurrence of epilepsy in autistic individuals, the most consistent variable was the presence of intellectual disability or cognitive impairment.^12^ Even in higher functioning individuals with ASD, one must question the etiologies leading to certain patterns of impairment. For example, in an individual with ASD and right hemisphere epilepsy is a language deficit associated with atypical language representation or is it a core feature of the ASD?

Children with epilepsy often have disrupted educational opportunities due to seizures, medical appointments, and hospital stays and may be excluded from school‐related activities due to teachers' or parents' fears of seizures, stigma, and difficulty with peer integration due to deficits in social cognition.^13^ In addition, these children may struggle with academic skill and knowledge attainment due to learning disabilities or the impact of other cognitive impairments (e.g., inattention, poor memory) on learning.^14^ All these factors can interfere with both formal and informal (but important) learning opportunities, which can have long‐term effects on cognitive development and performance in a neuropsychological assessment.

### 3.3 Epilepsy in adulthood

For those such as Ms. X whose epilepsy was diagnosed in childhood/adolescence, concern about the effects of chronic epilepsy and the risk of progressive cognitive decline has been longstanding. It has been shown that subsets of persons with active epilepsy may have a problematic cognitive course, or even cognitive decline, that is typically linked to a variety of risk factors (e.g., structural brain abnormalities, neuropathological findings, episodes of status epilepticus, number of lifetime generalized tonic–clonic seizures) and/or lack of factors that provide cognitive resilience (e.g., higher education, general mental ability, and socioeconomic status).^15^ Also, of concern and in need of monitoring are more transient and modifiable risk factors for cognitive difficulty, such as anti‐seizure medication (ASM) induced issues including polytherapy, toxicity, or specific ASM adverse effects (e.g., topiramate and language‐dependent skills).

These issues speak to how a “baseline assessment” of cognition and psychosocial factors, most optimally near the time of epilepsy onset, would be helpful. Such assessments serve to characterize how epilepsy and the wide variety of etiologies that may underlie the epilepsy (e.g., tumors, cerebral infections, cerebrovascular events, traumatic brain injury) impact one's mental status, establish an important baseline against which we may compare the patient going forward, and aid in pre‐surgical evaluation and prediction of potential costs and benefits of surgery.^2^ The findings can also be used to address any needed rehabilitation to maximize quality of life. The value of neuropsychology in the pre‐ and postoperative setting was demonstrated in detail for Ms. X. However, how Ms. X's cognitive profile may change with age given her complicated history of chronic childhood‐onset epilepsy, TBI, and epilepsy surgery remains a salient concern so ongoing neuropsychological monitoring would be advised.

Finally, as people age there is a predictable pattern of cognitive change occurring over decades. Some cognitive abilities (such as word knowledge) appear more resilient to age‐related decline, while other abilities (such as speeded processing and memory) appear more vulnerable to adverse aging effects.^16^ Adverse age‐related cognitive declines and cognitive disorders of aging (e.g., Alzheimer's disease) can be accelerated, or slowed, by health‐related factors (e.g., number, type(s), and treatment(s) for medical conditions), sociodemographic factors (e.g., education, work complexity, exposure to social disparities of health), and/or lifestyle patterns (e.g., diet, exercise, socialization, substance use), issues that have long been of concern for people with epilepsy.^17^, ^18^ These modifiable risk factors should be attended to in order to protect the cognitive and brain health of aging persons with epilepsy.

**Take‐home points**

- Consider the etiology of underlying the cause of epilepsy.
- Consider how age of seizure onset may result in a heterogeneity of cognitive outcomes.
- Think about the potential effects of comorbid neurodevelopmental disorders on cognition and other aspects of development.
- Epilepsy may disrupt formal and informal learning and social opportunities that can impact cognitive outcomes.
- Cognitive assessment should be a standard component of the initial evaluation for persons with late‐onset epilepsy and among individuals whose epilepsy onset earlier in life with standard prospective follow‐up to detect cognitive deterioration.

## 4 PSYCHIATRIC COMORBIDITIES

Psychiatric disorders are common in epilepsy, particularly anxiety, depression, psychosis, obsessive–compulsive disorder, and conduct problems.^19^ They can complicate the treatment of the epilepsy and adversely impact patient outcomes.^20^ Psychiatric disorder is considered by many to be an “essential” comorbidity of epilepsy^1^; that is, the brain network disease that causes the seizures also causes psychiatric symptoms. This shared network etiology is thought to give rise to the overlapping features of epilepsy and psychiatric disorders, such as cognitive impairments, fatigue, sleep changes, anhedonia, and physiological symptoms.^21^, ^22^ Cognitive impairments, in particular, can be a prodromal feature of both emerging seizures and psychiatric disturbance, fluctuate as part of an acute psychiatric state, and worsen over time with ongoing seizures and psychiatric disorder.^23^, ^24^

In the context of a neuropsychological examination, psychiatric comorbidities add complexity to case formulation. The presence of anxiety and depression can have a deleterious impact on both objective and subjective cognitive functioning. People with elevated levels of anxiety and depression have been shown to score significantly lower on measures of verbal intelligence, attention, visuomotor speed, executive function, and memory when compared to neurotypical controls.^25^, ^26^ Cognitive symptoms of mental illness, such as poor attention and concentration, memory impairment, rumination, impulsivity, and emotional dysregulation can distort or exaggerate the cognitive sequelae of epilepsy.^27^ Similarly, iatrogenic cognitive dysfunction can be linked to some psychiatric treatments: psychotropic medications can have side effects such as headache, nervousness, and sedation that compromise both test engagement and performance.^27^ Together, these issues may hinder testing and compromise the validity of a diagnostic neuropsychological consultation. Strategies for mitigating their impact are discussed.

### 4.1 Always ask about psychiatric history

Given that many people with epilepsy do not receive treatment for their psychiatric symptoms,^22^ directly asking about their psychiatric history may not always yield the necessary information. Clinicians may want to engage patients in conversation about the frequency of psychiatric disorders in people with epilepsy, to normalize the experience and create an environment that encourages them to share their experiences freely. Open‐ended and nonjudgmental questions can follow, such as “Have you or your loved ones ever felt like you needed help because of the way you were thinking, feeling, or behaving?” or “Can you tell me about the most challenging period in your life so far?”. Moreover, given that both epilepsy and psychiatric disorders remain stigmatized across many cultures, it is important to be educated around relevant cultural norms and beliefs when discussing mental health. These conversations should incorporate relevant illness‐related beliefs and language, which can often be sourced online from government and community‐based mental health services. For instance, in the case of Ms. X, her community of Spanish‐speaking adults in the United States may harbor stigma and misinformation about epilepsy and psychiatric disorder that needs to be considered as part of culturally competent care. She indicated that social effects of epilepsy were among the strongest reasons that she was motivated to pursue epilepsy surgery; conversely, this same community may also offer higher levels of family support and hold more positive views on taking care of mental well‐being as a means of holistic epilepsy care,^28^ which can be leveraged to benefit the patient's well‐being in the workup for surgery.

### 4.2 Maximizing patient engagement

Symptoms common to epilepsy and its psychiatric features like fatigue, anxiety, and attentional disturbances can hinder the patient's engagement with neuropsychological testing. Strategies for minimizing their impact include taking time to establish rapport; clarifying the importance of good effort to guide clinical decision‐making; allowing time to address anxieties; allowing rest breaks or multiple sessions to avoid overwhelming the patient and combat mental fatigue; and providing frequent positive encouragement.^29^ The use of formal effort testing can help evaluate whether psychometric results can be interpreted validly, as well as to inform interpretation of the broader test profile.

Careful consideration should be given to the timing of an assessment. In some cases, it may be best to defer neuropsychological evaluation until acute psychiatric symptoms have been treated to obtain the most reliable test performance. Alternatively, repeated assessments either over time or with differing tests can help to identify fluctuating cognitive effects of the psychiatric state from more stable cognitive effects of epilepsy.^29^

### 4.3 Principles for test interpretation

A particular challenge for neuropsychologists in epilepsy is delineating the cognitive effects of the underlying epilepsy versus the cognitive symptoms of mental illness. Psychiatric comorbidity may alter the psychometric profile and must be factored into the interpretation of the scores. For instance, people with epilepsy who have worse depressive symptoms have increasingly worse cognitive phenotypes than those without significant depressive symptomatology.^27^ If not carefully considered, there is a risk of diagnostic overshading, that is, the cognitive symptoms of the mental illness are erroneously attributed to the epilepsy, clouding the diagnostic formulation and resultant prognostic opinion. Making sense of this diagnostic puzzle relies on the clinician interpreting the test scores within the context of the clinical history, patient complaints, and testing behavior.

### 4.4 Distinguishing cognitive impairments attributable to epilepsy versus psychiatric conditions

The key question for clinicians to ask is as follows: What cognitive impairments can be reasonably attributed to the person's epilepsy, versus their psychiatric condition? This requires understanding what cognitive dysfunction is expected in the context of the patient's epilepsy syndrome, pathology, or hypothesized epileptogenic zone, as well as the cognitive sequelae of the relevant psychiatric condition and its treatment. In the case of Ms. X, the presumed epileptogenic zone in the right temporal lobe together with hippocampal pathology in the left hemisphere can reasonably account for her globally reduced memory. However, her reported social stigma, bereavement following the death of her grandmother, and poor epilepsy‐related quality of life affected her emotional well‐being. If the evaluating neuropsychologist had concerns that these psychosocial challenges were negatively impacting her mood enough to influence her cognitive test performance, then they may have moved to repeat psychometric testing once stressors settled. In cases where there are serious doubts, taking this more conservative approach may help clarify the impact of mood on higher‐order aspects of her cognition like attention and executive function that can exacerbate memory difficulties.

### 4.5 Behavioral indicators in test interpretation

Test interpretation is also often informed by patient approach, errors, and behavior during the evaluation. An anxious patient, for instance, may “freeze” periodically during timed tasks like verbal fluency or tasks with a lot of information to process like list learning or prose recall; in these cases, the patient may appear overtly stressed or overwhelmed, “blank out,” or produce fluctuating output. Patterns such as failing easy tasks but succeeding on more challenging ones may also be indicative of reduced test engagement. In contrast, patients doing poorly on the same tasks secondary to epilepsy‐related sedation or slow processing speed often instead seem calm and methodical in their approach to the task and produce consistently reduced output. Depressed patients may similarly work slowly, but appear disengaged, self‐criticize, and give up easily once tasks become challenging.

This approach to test interpretation balances the “art” of clinical practice with the availability of scientific evidence around cognitive phenotypes specific to different epilepsies.^30^ As with any balancing act, it has limitations. Chiefly, it is not possible to reliably attribute all cognitive dysfunction to epilepsy (including medication‐related effects) versus psychiatric comorbidities when many features overlap. For instance, overgeneral autobiographical memory is a trait‐like vulnerability factor for unipolar depression that is also commonly impaired in focal epilepsies^31^, ^32^; at present, however, it is often not possible to distinguish which of the two mechanisms is responsible for an autobiographical memory impairment when both are present. Instead, clinicians need to look for other supportive features of a cognitive phenotype of epilepsy; for instance, autobiographic memory dysfunction in mesial temporal lobe epilepsies is commonly accompanied by features not canonically seen in depression, like accelerated long‐term forgetting.^33^

### 4.6 Strategies for differentiating cognitive impairments

To separate out any state‐dependent cognitive effects of comorbid psychopathology, other strategies include retesting the patient when their mental state has improved and obtaining a timeline of when the cognitive issues emerged relative to the epilepsy and psychiatric disorder. The latter may also incorporate a collateral history from a caregiver or family member, as the patient's own subjective cognitive concerns are sometimes a stronger marker of psychological distress than objective neuropsychological deficits or neurological pathology, and as a result may not align with the known cognitive sequelae of the neurological disease.^34^, ^35^ Moreover, the nature of the cognitive complaints offered by people with epilepsy may provide clues as to whether they stem from depression, anxiety, or organic cognitive disorder, with anxious and depressed mood most likely to give rise to memory complaints mediated by executive functions verses impoverished autobiographic memory, respectively.^36^ Performance and symptom validity testing are also important to include in test batteries to help increase clinician confidence in the validity and reliability of obtained test results.

Clinicians are also encouraged to familiarize themselves with the evolving theoretical models and diagnostic approaches for functional cognitive disorder^37^, ^38^ which is distinct from functional neurological disorder and takes the form of disabling cognitive concerns that are typically incongruent with the patient's normal test results, everyday functioning, and/or conversational abilities. This is because neuropsychological management for a person with epilepsy and functional cognitive disorder will be significantly different than for the patient with cognitive impairments attributable to epilepsy or comorbid psychiatric disorder.^38^

### 4.7 Implications for treatment

Once a clinical opinion has been formulated, it can be used to inform medical and neuropsychological treatment planning. As a first step, it is crucial to elicit the patient's treatment goals, understand their subjective concerns, and involve them in goal‐setting to ensure alignment between test data interpretation and their broader treatment aspirations. In the case of Ms. X, for instance, her bilateral hippocampal pathology presents the potential risk of postoperative amnesia (a relatively low risk given the results of the memory Wada testing) as well as postoperative mood disorder.^39^ Combined with her mildly elevated depressive symptoms, she may have been at an increased risk of a poor surgical outcome in terms of increased risk of seizure recurrence, potential memory decline, and a postoperative mood disorder (i.e., “triple jeopardy”). This could render the patient significantly worse off after surgery, which could undermine her goals to improve her social life and overall level of functioning.^2^ This possibility was discussed with Ms. X thoroughly in pre‐surgical neuropsychological counseling, and she assured the team that much of her low mood was situational and she was motivated to pursue surgery in hopes of seizure reduction positively impacting her psychosocial situation. In her case, with a reduction in seizure activity, her depression symptoms dramatically improved.

**Take‐home points**

- Always ask about psychiatric history and symptoms.
- Psychiatric comorbidities can alter neuropsychological test profiles and must be factored into score interpretation.
- Consider: What is the patient likely to experience cognitively because of (i) their epilepsy versus (ii) their psychiatric condition?

## 5 PSYCHOSOCIAL ISSUES

**FIGURE 1:**
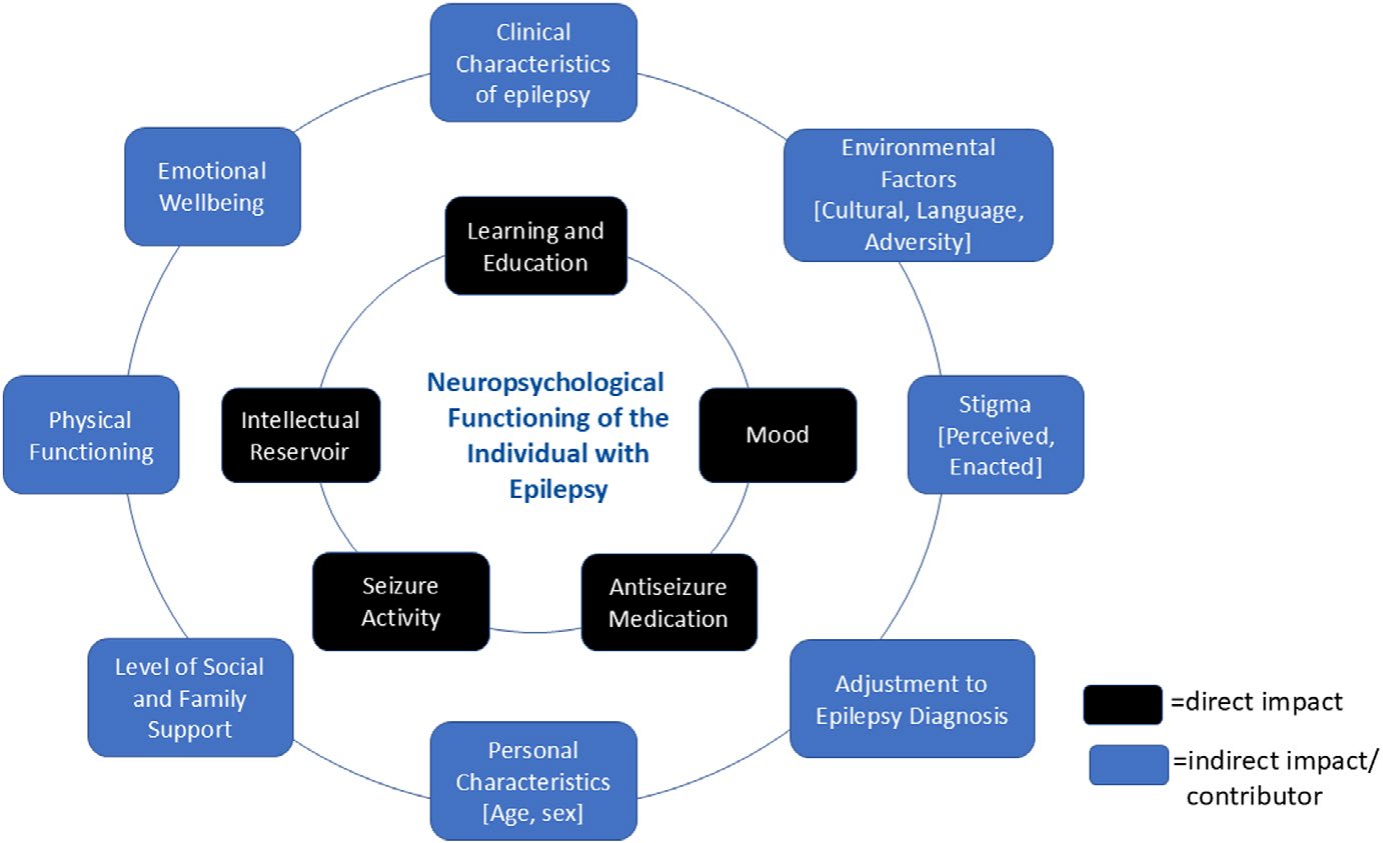
Influences on neuropsychological functioning in people with epilepsy.

It is important to consider that psychosocial factors significantly contribute to the neuropsychological impact of epilepsy. While clinical factors such as epilepsy‐related and psychiatric comorbidities are considered to have a direct effect, psychosocial factors have both direct and indirect influences on neuropsychological consequences and outcomes (see Figure 1). Ms. X was at especial risk for susceptibility to psychosocial disorder as it has been shown that the combined psychosocial impacts arising from TBI and epilepsy are beyond that of either condition alone.^40^

Ms. X encountered numerous challenging circumstances and life‐altering events, including early onset seizures, long‐term use of anti‐seizure medications, immigrating to a new country, pursuing education in a new language, suffering a TBI with consequent compromised cognitive functioning, living alone, experiencing bereavement following the loss of a close family member, undergoing early menopause, being underemployed with potential financial constraints, and facing social stigma.

This section highlights those salient psychosocial factors that have an impact, including adjustment, stigma, social isolation, and life adversity. Lastly, the impact of cultural and language diversity on the assessment of neuropsychological functioning needs to be considered.

### 5.1 Adjustment

The challenges faced by Ms. X and related social stigma impacted her mood and increased the burden of epilepsy. Studies have documented the deleterious effect of epilepsy on all aspects of life including education, employment, financial status, relationships, driving, parenting, and social activities.^41^ Consequently, epilepsy has been described as an “intrusive illness” that disrupts the individual's ability to engage in the usual activities of life.^42^ Responses to a diagnosis of epilepsy can range from acceptance to feelings of hopelessness and helplessness.^43^, ^44^ While adjustment to the diagnosis of epilepsy may not directly influence cognitive functioning, the secondary effects of failing to adjust may be linked to a range of symptoms, including anxiety, sleep difficulties, and low mood which can have an indirect impact on test performance.^45^

### 5.2 Stigma of epilepsy

A central feature of epilepsy is its stigmatizing nature. Myths and misconceptions about epilepsy, both historical and current, serve to create and maintain this stigma, and may lead to discrimination despite attempts to better educate the general population about epilepsy and seizures.^46^ Ms. X herself expressed feeling significant social stigma, and it was a driver of her motivation for surgery. It is unlikely that feeling stigmatized will directly impact her cognitive performance; however, there is sufficient evidence to suggest that high levels of perceived stigma place an individual at risk of anxiety and depression^23^ which can in turn influence cognitive functioning as detailed above.

### 5.3 Social isolation

Ms. X lived alone, had never married, and was childless. It was the impact of her seizures on her social life that prompted her to seek surgical treatment. Social isolation is a contributor to the psychosocial burden experienced by people with epilepsy and may be a consequence of both seizure and social factors. The unpredictable nature of seizures, for example, makes for planning and scheduling activities difficult and can lead to increased levels of anxiety and fear in both people with epilepsy and others around them. Stigma and other cross‐cultural factors are also suggested to contribute.^47^, ^48^, ^49^ Consequently, people with epilepsy may withdraw from others or avoid activities and, over time, become increasingly socially isolated.^50^

Social isolation has a bidirectional association with psychopathology such as anxiety and depressive disorders^49^ while effective social support and positive interactions can help reduce maladjustment to an epilepsy diagnosis.^50^ Exploring psychosocial history and the availability of social support during the clinical interview helps the clinician make inferences about psychosocial contributions to neuropsychological presentation and plays a role in managing expectations for outcomes, as demonstrated in Ms. X's case.

### 5.4 Adversity

Ms. X experienced cognitive difficulties after her TBI, forcing her to leave school and work as an assembler in a relatively low‐demand job. She reported mood issues while coping with this adverse situation. Epilepsy is, itself, a life‐altering, adverse situation. Beyond seizures, adverse environmental factors of low socioeconomic status (SES) and living in deprived environments affect neuropsychological functioning in children and adults.^51^, ^52^, ^53^ Lack of educational opportunities, limited access to health care, poor nutrition, and increased medical comorbidities are potential mechanisms affecting cognition and mood.^51^ People with epilepsy from low SES backgrounds may also have poor test‐taking sophistication, and the lack of “test wiseness” may lead to decreased motivation and suboptimal test performance. As poor outcomes and greater cognitive impairments after epilepsy surgery are associated with low SES,^54^ it is important to observe behavior carefully during the testing session and use appropriate normative data to try and determine how much of a patient's background may be contributing to the clinical picture beyond clinical‐epilepsy‐related factors themselves.

### 5.5 Challenges of cultural and language diversity

Cultural and language diversity create unique challenges in the assessment of people with epilepsy. In Ms. X's case, both factors were considered for test selection and finding appropriate normative comparisons. Although she was not evaluated by someone with a similar linguistic and cultural background, the neuropsychologist had expertise in epilepsy and complex pre‐surgical evaluations, and the use of certified Spanish‐language interpreters and psychometrists enhanced the validity and reliability of her neuropsychological examination.^55^

Bilingual and multilingual patients are challenged by two factors when undertaking linguistic tests: interference and reduced frequency of use.^56^ Interference refers to the extent to which one language needs to be inhibited to produce another, and subsequently, stronger inhibitory control is needed when accessing a nondominant language.^56^ When people speak more than one language, frequency of use is reduced in both languages and thus performance is typically reduced regardless of the language assessed.^57^, ^58^ These influences may result in reduced performance across all spoken languages, including the dominant language, impacting the recognition of difficult words, tip‐of‐the‐tongue errors, naming speed, and confrontation naming performance.^59^, ^60^, ^61^ For these reasons, clinicians should use norms derived from bilingual individuals when available and consider language background when interpreting results.

Cognition is the product of a dynamic interplay of biological, socioeconomic, and cultural determinants, yet the science and practice of neuropsychology have largely ascribed a universal approach to cognition.^62^ This approach leads to inadequate science, erroneous racial and ethnic generalizations, and inappropriate use of tests across diverse populations. Although there has been a lack of appropriately standardized and culturally relevant neuropsychological tests, recent efforts have been made to ameliorate this situation and neuropsychologists should familiarize themselves with appropriate options.^63^

Culture determines internal, subjective representations like feelings, beliefs, attitudes, and values as well as behavioral aspects like ways of relating to others. These factors are shaped by experience and learning and lead to specific social norms that impact on a patient's interaction with the clinician, approach to tests, and test performance itself. Evidence shows that cultural background differentially affects the performance of verbal and non‐verbal tests.^64^ For instance, speakers of different languages performed differently on verbal fluency tasks, with Spanish speakers generating fewer words in phonemic fluency tasks compared to English speakers, but performing similarly in semantic fluency tasks. In addition, the approach to and strategy used in non‐verbal tests have shown to differ across cultures. Unique approaches to and performance on cognitive tests are primarily underscored by differing values and meanings, modes of knowing, and conventions of communication.^65^

### 5.6 Influence of healthcare disparities in epilepsy

Beyond the clinical features, Ms. X's case underscores the importance of considering the broader social and structural context in which epilepsy care occurs—particularly the persistent disparities that disproportionately affect racial/ethnic and linguistic minorities, as well as individuals with low socioeconomic status.^66^ For example, in the United States, Black and Hispanic patients have reduced access to specialized care, higher reliance on emergency and generalist services, increased hospitalization rates, and lower rates of specialist visits—even when controlling for other variables.^67^, ^68^ Access to diagnostics like video‐EEG remains uneven, with barriers persisting for publicly insured or uninsured individuals despite proximity to epilepsy centers.^69^ These disparities extend to surgical care, where Black, Hispanic, and non‐English‐speaking patients are significantly less likely to receive surgical evaluation or resective epilepsy surgery, due to a combination of fear, limited access, communication challenges, mistrust, and low social support.^70^ Expectations around surgical outcomes also vary by race, with white patients often prioritizing cognitive, educational, and employment goals, while nonwhite patients more frequently emphasize driving.^71^

### 5.7 Implications of psychosocial aspects for neuropsychological testing

Diverse psychosocial determinants of health need to be understood as they may impact neuropsychological test performance directly, in terms of selection of culturally appropriate tests and language administration; and indirectly by other factors that influence mood and behaviors during testing.

**Take‐home points**

- Explore psychosocial factors like cultural beliefs, language(s), indicators of adversity, experience of stigma, and adjustment issues as part of an evaluation.
- Recognize that these factors may influence test‐taking attitudes, behaviors, and mood, thereby impacting test performance.
- Make efforts to address these issues during testing (cultural suitability of tests, appropriate language of administration, rapport and reassurance during testing) to optimize performance.
- Be aware that systemic healthcare disparities—especially in access to specialized and surgical epilepsy care—may shape a patient's diagnostic journey, expectations, and engagement with treatment, all of which may affect neuropsychological evaluation and treatment planning.

## 6 CHALLENGES OF NEUROPSYCHOLOGICAL EVALUATIONS IN LOW‐ AND MIDDLE‐INCOME COUNTRIES (LMIC)

Neuropsychological services in LMIC face challenges of limited resources and poor understanding of neuropsychology resulting in a “evaluation gap” for neuropsychological testing in epilepsy. In most LMIC, specialty health services are available only in major urban centers with persons from underserved rural areas not having an opportunity to access the same. There is low priority for developing standardized tools, few qualified neuropsychologists, decreased opportunities for specialty training, absence of guidelines, and considerable heterogeneity in test selection and lack of appropriate normative data.^72^ Hence, empirical assessment is often replaced by non‐standardized screening by clinicians who assess cognitive abilities through observation‐based subjective reports^73^.

Test performance is affected not only by the cultural and linguistic diversity (as indicated in the sections above), but also by low literacy and variable levels of quality of education.^73^ Discriminative abilities of tests are reported to be lower in low literacy patients, and the clinician has to be aware of certain common strategies spontaneously used by people with low literacy during the testing. For example, it is common for them to use fingers to count in a serial subtraction task or repeat words aloud after the examiner in encoding the word list and these strategies may need to be allowed in low literacy groups.^74^ Test instructions may need to be elaborated, scoring rules changed, and the results interpreted in the context of multiple factors that can impact cognitive testing in LMIC. Local norms need to be collected reflecting these alterations.

A major challenge in LMIC is that patients and families from low literacy and rural backgrounds often lack awareness and understanding of the association of cognitive deficits with epilepsy, resulting in devaluation of the testing procedure. The lack of “test wiseness” can lead to suboptimal performance on tests and the need for “cognitive conversations” before conducting an evaluation to educate people on cognitive deficits and what testing procedures entail and explain the value of the testing.^75^

Capacity building of LMIC neuropsychologists by offering remote, virtual training may be the way forward. Teleneuropsychology is also being explored as a method to offer at least basic level testing protocols in such settings.^75^, ^76^

**Take‐home points**

- Consider all the standardization details of the test—adaptations, translations, and education‐stratified normative data.
- Think about the modifications in instructions, scoring methods, and pre‐testing psychoeducation to ensure “fair” evaluations.
- Be aware of strategies used by low literacy groups and do not disallow the same.
- Development of simple test protocols that can be used in tele assessments may in the future facilitate access to the neuropsychological evaluations for a large population of people with epilepsy living in remote, unreached areas.

## 7 CONCLUSION

This review has highlighted the complexity of the neurodevelopmental, psychiatric, and psychosocial factors that can affect neuropsychological test performance. These factors highlight the need for expert knowledge among neuropsychologists in the field of epilepsy, as such knowledge shapes the clinician's ability to make appropriate diagnoses and recommendations. It is incumbent on practitioners to recognize and consider these issues, incorporate them into their judgments and to acknowledge the limits such issues place on their formulations. Furthermore, the complexity of problem‐solving and information gathering that is needed indicate that appropriate lengths of time need to be allocated to the assessment of people with epilepsy. These factors and their implications should feature prominently in the feedback given to patients, their caregivers, and referring providers.

## CONFLICT OF INTEREST STATEMENT

None of the authors have conflicts of interest to declare.

**Test yourself**

1. Modifiable risk factors that can impact on neuropsychological function include all but which of the following?
  - Sociodemographic factors
  - Side effects of anti‐seizure medications
  - Underlying brain pathology
  - Lifestyle patterns
2. What is a key challenge for neuropsychologists when evaluating the cognitive profile of a person with epilepsy and comorbid depression?
  - Distinguishing between the cognitive effects of epilepsy versus the mental illness
  - Determining the cause of the depression
  - Identifying the specific location of the epileptogenic zone
  - Ensuring patients follow the treatment plan accurately
3. How do psychosocial factors influence neuropsychological functioning in epilepsy?
  - They only have indirect effects.
  - Stigma affects self‐perception but not cognition.
  - Social isolation can lead to anxiety and depression, impacting cognition.
  - Standardized neuropsychological tests remove cultural and linguistic biases.

Answers may be found in the *supporting information*.

## Supporting information


Appendix S1.

Appendix S2.

Figures S1–S2.

## Data Availability

Data sharing is not applicable to this article as no new data were created or analyzed in this study.

## References

[1] Wilson SJ , Baxendale S . The new approach to classification: rethinking cognition and behavior in epilepsy. Epilepsy Behav. 2014;41:307–310. 25440828 10.1016/j.yebeh.2014.09.011

[2] Baxendale S , Wilson SJ , Baker GA , Barr W , Helmstaedter C , Hermann BP , et al. Indications and expectations for neuropsychological assessment in epilepsy surgery in children and adults: report of the ILAE neuropsychology task force diagnostic methods commission: 2017–2021 neuropsychological assessment in epilepsy surgery. Epileptic Disord. 2019;21(3):221–234. 31262718 10.1684/epd.2019.1065

[3] Wilson SJ , Baxendale S , Barr W , Hamed S , Langfitt J , Samson S , et al. Indications and expectations for neuropsychological assessment in routine epilepsy care: report of the ILAE neuropsychology task force, diagnostic methods commission, 2013‐2017. Epilepsia. 2015;56(5):674–681. 10.1111/epi.12962 25779625

[4] Cherner M , Marquine MJ , Umlauf A , Morlett Paredes A , Rivera Mindt M , Suárez P , et al. Neuropsychological norms for the U.S.‐Mexico border region in Spanish (NP‐NUMBRS) project: methodology and sample characteristics. Clin Neuropsychol. 2021;35(2):253–268. 10.1080/13854046.2019.1709661 32319851 PMC7894577

[5] Maj M , D'Elia L , Satz P , Janssen R , Zaudig M , Uchiyama C , et al. Evaluation of two new neuropsychological tests designed to minimize cultural bias in the assessment of HIV‐1 seropositive persons: a WHO study. Arch Clin Neuropsychol. 1993;8(2):123–135. 14589670

[6] Witt JA , Elger CE , Helmstaedter C . Impaired verbal fluency under topiramate–evidence for synergistic negative effects of epilepsy, topiramate, and polytherapy. Eur J Neurol. 2013;20(1):130–137. 22827489 10.1111/j.1468-1331.2012.03814.x

[7] Baxendale S . IQ and ability across the adult life span. Appl Neuropsychol. 2011;18(3):164–167. 10.1080/09084282.2011.595442 21846215

[8] Stasenko A , Schadler A , Kaestner E , Reyes A , Díaz‐Santos M , Połczyńska M , et al. Can bilingualism increase neuroplasticity of language networks in epilepsy? Epilepsy Res. 2022;182:e06893. 10.1016/j.eplepsyres.2022.106893 PMC9050932 35278806

[9] Balestrini S , Arzimanoglou A , Blümcke I , Scheffer IE , Wiebe S , Zelano J , et al. The aetiologies of epilepsy. Epileptic Disord. 2021;23(1):1–16. 33720020 10.1684/epd.2021.1255

[10] Hermann BP , Zhao Q , Jackson DC , Jones JE , Dabbs K , Almane D , et al. Cognitive phenotypes in childhood idiopathic epilepsies. Epilepsy Behav. 2016;61:269–274. 27442497 10.1016/j.yebeh.2016.05.013 PMC4998056

[11] Auvin S , Wirrell E , Donald KA , Berl M , Hartmann H , Valente KD , et al. Systematic review of the screening, diagnosis, and management of ADHD in children with epilepsy. Consensus paper of the task force on comorbidities of the ILAE pediatric commission. Epilepsia. 2018;59(10):1867–1880. 30178479 10.1111/epi.14549

[12] Zarakoviti E , Shafran R , Skuse D , McTague A , Batura N , Palmer T , et al. Factors associated with the occurrence of epilepsy in autism: a systematic review. J Autism Dev Disord. 2023;53(10):3873–3890. 35904650 10.1007/s10803-022-05672-2 PMC10499929

[13] Stewart E , Lah S , Smith ML . Patterns of impaired social cognition in children and adolescents with epilepsy: the borders between different epilepsy phenotypes. Epilepsy Behav. 2019;100:106146. 10.1016/j.yebeh.2019.01.031 30894295

[14] Reilly C , Neville BG . Academic achievement in children with epilepsy: a review. Epilepsy Res. 2011;97(1–2):112–123. 21924868 10.1016/j.eplepsyres.2011.07.017

[15] Thompson PJ , Duncan JS . Cognitive decline in severe intractable epilepsy. Epilepsia. 2005;46(11):1780–1787. 10.1111/j.1528-1167.2005.00279.x 16302858

[16] Salthouse TA . Trajectories of normal cognitive aging. Psychol Aging. 2019;34(1):17–24. 10.1037/pag0000288 30211596 PMC6367038

[17] Kobau R , Zahran H , Thurman DJ , Zack MM , Henry TR , Schachter SC , et al. Epilepsy surveillance among adults‐‐19 states, behavioral risk factor surveillance system, 2005. MMWR Surveill Summ. 2008;57(6):1–20. 10.1212/WNL.0000000000011080 18685554

[18] Livingston G , Huntley J , Liu KY , Costafreda SG , Selbæk G , Alladi S , et al. Dementia prevention, intervention, and care: 2024 report of the lancet standing commission. Lancet. 2024;404(10452):572–628. 10.1016/S0140-6736(24)01296-0 39096926

[19] Tellez‐Zenteno JF , Patten SB , Jetté N , Williams J , Wiebe S . Psychiatric comorbidity in epilepsy: a population‐based analysis. Epilepsia. 2007;48(12):2336–2344. 17662062 10.1111/j.1528-1167.2007.01222.x

[20] Fazel S , Wolf A , Långström N , Newton CR , Lichtenstein P . Premature mortality in epilepsy and the role of psychiatric comorbidity: a total population study. Lancet. 2013;382:1646–1654. 23883699 10.1016/S0140-6736(13)60899-5 PMC3899026

[21] Roberts‐West L , Vivekananda U , Baxendale S . Anhedonia in epilepsy. Epilepsy Behav. 2023;140:108966. 36443164 10.1016/j.yebeh.2022.108966

[22] Rayner G , Jackson GD , Wilson SJ . Two distinct symptom‐based phenotypes of depression in epilepsy yield specific clinical and etiological insights. Epilepsy Behav. 2016;64:336–344. 27473594 10.1016/j.yebeh.2016.06.007

[23] Allott K . Staging of cognition in psychiatric illness. In: McGorry PD , Hickie IB , Yung AR , Pantelis C , Bertolote J , editors. Clinical staging in psychiatry: making diagnosis work for research and treatment. Cambridge: Cambridge University Press; 2019. p. 40–171.

[24] Pugh R , Vaughan DN , Jackson GD , Ponsford J , Tailby C . Cognitive and psychological dysfunction is present after a first seizure, prior to epilepsy diagnosis and treatment at a first seizure clinic. Epilepsia Open. 2024;9(2):717–726. 38319041 10.1002/epi4.12909 PMC10984291

[25] Riso LP , du Toit PL , Blandino JA , Penna S , Dacey S , Duin JS , et al. Cognitive aspects of chronic depression. J Abnorm Psychol. 2003;112(1):72–80. 12653415

[26] Yeni K , Tulek Z , Simsek OF , Bebek N . Relationships between knowledge, attitudes, stigma, anxiety and depression, and quality of life in epilepsy: a structural equation modeling. Epilepsy Behav. 2018;85:212–217. 30032810 10.1016/j.yebeh.2018.06.019

[27] Bingaman N , Ferguson L , Thompson N , Reyes A , McDonald CR , Hermann BP , et al. The relationship between mood and anxiety and cognitive phenotypes in adults with pharmacoresistant temporal lobe epilepsy. Epilepsia. 2023;64:3331–3341. 37814399 10.1111/epi.17795 PMC11470599

[28] Sirven JI , Lopez RA , Vazquez B , Van Haverbeke P . Que es la Epilepsia? Attitudes and knowledge of epilepsy by Spanish‐speaking adults in the United States. Epilepsy Behav. 2005;7(2):259–265. 16054871 10.1016/j.yebeh.2005.04.015

[29] Calev A , Preston T , Samuel S , Gorton GE . Clinical neuropsychological assessment of psychiatric disorders. In: Calev A , editor. Assessment of neuropsychological functions in psychiatric disorders. Washington, DC: American Psychiatric Association; 1999. p. 1–32.

[30] McDonald CR , Busch RM , Reyes A , Arrotta K , Barr W , Block C , et al. Development and application of the international classification of cognitive disorders in epilepsy (IC‐CoDE): initial results from a multi‐center study of adults with temporal lobe epilepsy. Neuropsychology. 2023;37(3):301–314. 35084879 10.1037/neu0000792 PMC9325925

[31] Hitchcock C , Rodrigues E , Rees C , Gormley S , Dritschel B , Dalgleish T . Misremembrance of things past: depression is associated with difficulties in the recollection of both specific and categoric autobiographical memories. Clin Psychol Sci. 2019;7:693–700. 32655985 10.1177/2167702619826967 PMC7324083

[32] Rayner G , Jackson GD , Wilson SJ . Mechanisms of memory impairment in epilepsy depend on age at disease onset. Neurology. 2016;87:1642–1649. 27638925 10.1212/WNL.0000000000003231 PMC5085077

[33] Baker J , Savage S , Milton F , Butler C , Kapur N , Hodges J , et al. The syndrome of transient epileptic amnesia: a combined series of 115 cases and literature review. Brain Commun. 2021;3:fcab038. 33884371 10.1093/braincomms/fcab038 PMC8047097

[34] Mücke FJ , Hendriks MP , Bien CG , Grewe P . Discrepancy between subjective and objective memory change after epilepsy surgery: relation with seizure outcome and depressive symptoms. Front Neurol. 2022;13:855664. 35937068 10.3389/fneur.2022.855664 PMC9355315

[35] Rayner G , Wrench JM , Wilson SJ . Differential contributions of objective memory and mood to subjective memory complaints in refractory focal epilepsy. Epilepsy Behav. 2010;19:359–364. 20947435 10.1016/j.yebeh.2010.07.019

[36] Trend C , Puntambekar I , Baxendale S . Subjective memory complaints in people with epilepsy: are there “signature” complaints associated with anxiety and depression? Epilepsia Open. 2025. 10.1002/epi4.70027 PMC12163533 40119880

[37] Silverberg ND , Rush BK . Neuropsychological evaluation of functional cognitive disorder: a narrative review. Clin Neuropsychol. 2024;38(2):302–325. 37369579 10.1080/13854046.2023.2228527

[38] Cabreira V , McWhirter L , Carson A . Functional cognitive disorder: diagnosis, treatment, and differentiation from secondary causes of cognitive difficulties. Neurol Clin. 2023;41(4):619–633. 37775194 10.1016/j.ncl.2023.02.004

[39] Wrench JM , Rayner G , Wilson SJ . Profiling the evolution of depression after epilepsy surgery. Epilepsia. 2011;52(5):900–908. 21426325 10.1111/j.1528-1167.2011.03015.x

[40] Semple BD , Zamani A , Rayner G , Shultz SR , Jones NC . Affective, neurocognitive and psychosocial disorders associated with traumatic brain injury and post‐traumatic epilepsy. Neurobiol Dis. 2019;123:27–41. 10.1016/j.nbd.2018.07.018 30059725 PMC6348140

[41] Baker GA , Jacoby A , Buck D , Stalgis C , Monnet D . Quality of life of people with epilepsy: a European study. Epilepsia. 1997;38(3):353–362. 9070599 10.1111/j.1528-1157.1997.tb01128.x

[42] Poochikian‐Sarkissian S , Sidani S , Wennberg R , Devins GM . Seizure freedom reduces illness intrusiveness and improves quality of life in epilepsy. Can J Neurol Sci. 2008;35(3):280–286. 18714794 10.1017/s0317167100008842

[43] Räty LK , Wilde‐Larsson BM . Patients' perceptions of living with epilepsy: a phenomenographic study. J Clin Nurs. 2011;20(13–14):1993–2002. 21457376 10.1111/j.1365-2702.2010.03572.x

[44] Wilson SJ , Rayner G , Pieters J . Positive illusions determine quality of life in drug‐resistant epilepsy. Epilepsia. 2020;61(3):539–548. 10.1111/epi.16455 32108938

[45] Ogawa M , Fujikawa M , Jin K , Kakisaka Y , Ueno T , Nakasato N . Acceptance of disability predicts quality of life in patients with epilepsy. Epilepsy Behav. 2021;120:107979. 33962248 10.1016/j.yebeh.2021.107979

[46] Jacoby A , Snape D , Baker GA . Epilepsy and social identity: the stigma of a chronic neurological disorder. Lancet Neurol. 2005;4(3):171–178. 15721827 10.1016/S1474-4422(05)01014-8

[47] Baker GA . The psychosocial burden of epilepsy. Epilepsia. 2002;43(Suppl 6):26–30. 10.1046/j.1528-1157.43.s.6.12.x 12190975

[48] Mlinar S , Petek D , Cotič Ž , Mencin Čeplak M , Zaletel M . Persons with epilepsy: between social inclusion and marginalisation. Behav Neurol. 2016;2016:2018509. 27212802 10.1155/2016/2018509 PMC4861793

[49] Zhong R , Zhang H , Chen Q , Guo X , Han Y , Lin W . Social isolation and associated factors in Chinese adults with epilepsy: a cross‐sectional study. Front Neurol. 2021;12:813698. 35087477 10.3389/fneur.2021.813698 PMC8787157

[50] Saada F , Wang ZS , Bautista RE . In focus: the everyday lives of families of adult individuals with epilepsy. Epilepsy Behav. 2015;50:10–13. 26093217 10.1016/j.yebeh.2015.05.041

[51] Busch RM , Dalton JE , Jehi L , Ferguson L , Krieger NI , Struck AF , et al. Association of neighborhood deprivation with cognitive and mood outcomes in adults with pharmacoresistant temporal lobe epilepsy. Neurology. 2023;100(23):e2350–e2359. 37076308 10.1212/WNL.0000000000207266 PMC10256132

[52] Hermann B , Conant LL , Cook CJ , Hwang G , Garcia‐Ramos C , Dabbs K , et al. Network, clinical and sociodemographic features of cognitive phenotypes in temporal lobe epilepsy. Neuroimage Clin. 2020;27:102341. 32707534 10.1016/j.nicl.2020.102341 PMC7381697

[53] Huber R , Weber P . Is there a relationship between socioeconomic factors and prevalence, adherence and outcome in childhood epilepsy? A systematic scoping review. Eur J Paediatr Neurol. 2022;38:1–6. 35248913 10.1016/j.ejpn.2022.01.021

[54] Baxendale S , Heaney D . Socioeconomic status, cognition, and hippocampal sclerosis. Epilepsy Behav. 2011;20(1):64–67. 21130698 10.1016/j.yebeh.2010.10.019

[55] Nielsen TR , Franzen S , Watermeyer T , Jiang J , Calia C , Kjærgaard D , et al. Interpreter‐mediated neuropsychological assessment: clinical considerations and recommendations from the European consortium on Cross‐cultural neuropsychology (ECCroN). Clin Neuropsychol. 2024;38(8):1775–1805. 10.1080/13854046.2024.2335113 38588670

[56] Gollan TH , Montoya RI , Cera C , Sandoval TC . More use almost always means a smaller frequency effect: aging, bilingualism, and the weaker links hypothesis. J Mem Lang. 2008;58(3):787–814. 19343088 10.1016/j.jml.2007.07.001 PMC2409197

[57] Oldfield RC , Wingfield A . Response latencies in naming objects. Q J Exp Psychol. 1965;17(4):273–281. 5852918 10.1080/17470216508416445

[58] Scarborough DL , Cortese C , Scarborough HS . Frequency and repetition effects in lexical memory. J Exp Psychol Hum Percept Perform. 1977;3(1):1–17.

[59] Gollan TH , Acenas LA . What is a TOT? Cognate and translation effects on tip‐of‐the‐tongue states in Spanish‐English and Tagalog‐English bilinguals. J Exp Psychol Learn Mem Cogn. 2004;30(1):246–269. 14736310 10.1037/0278-7393.30.1.246

[60] Gollan TH , Brown AS . From tip‐of‐the‐tongue (TOT) data to theoretical implications in two steps: when more TOTs means better retrieval. J Exp Psychol Gen. 2006;135(3):462–483. 16846276 10.1037/0096-3445.135.3.462

[61] Ivanova I , Costa A . Does bilingualism hamper lexical access in speech production? Acta Psychol. 2008;127(2):277–288. 10.1016/j.actpsy.2007.06.003 17662226

[62] Rivera Mindt M , Arentoft A , Kubo Germano K , D'Aquila E , Scheiner D , Pizzirusso M , et al. Neuropsychological, cognitive, and theoretical considerations for evaluation of bilingual individuals. Neuropsychol Rev. 2008;18(3):255–268. 18841477 10.1007/s11065-008-9069-7 PMC2652412

[63] Franzen S , Watermeyer TJ , Pomati S , Papma JM , Nielsen TR , Narme P , et al. Cross‐cultural neuropsychological assessment in Europe: position statement of the European consortium on cross‐cultural neuropsychology (ECCroN). Clin Neuropsychol. 2022;36(3):546–557. 10.1080/13854046.2021.1981456 34612169

[64] Rosselli M , Ardila A , Salvatierra J , Marquez M , Matos L , Weekes VA . A cross‐linguistic comparison of verbal fluency tests. Int J Neurosci. 2002;112(6):759–776. 12325314 10.1080/00207450290025752

[65] Greenfield PM . You can't take it with you: why ability assessments don't cross cultures. Am Psychol. 1997;52:1115–1124.

[66] Burneo JG , Jette N , Theodore W , Begley C , Parko K , Thurman DJ , et al. Disparities in epilepsy: report of a systematic review by the north American Commission of the International League against Epilepsy. Epilepsia. 2009;50(10):2285–2295. 10.1111/j.1528-1167.2009.02282.x 19732134 PMC3181115

[67] Begley CE , Basu R , Reynolds T , Lairson DR , Dubinsky S , Newmark M , et al. Sociodemographic disparities in epilepsy care: results from the Houston/New York City health care use and outcomes study. Epilepsia. 2009;50(5):1040–1050. 10.1111/j.1528-1167.2008.01898.x 19054413

[68] Kroner BL , Fahimi M , Kenyon A , Thurman DJ , Gaillard WD . Racial and socioeconomic disparities in epilepsy in the District of Columbia. Epilepsy Res. 2013;103(2–3):279–287. 10.1016/j.eplepsyres.2012.07.005 22858309 PMC4608437

[69] Schiltz NK , Koroukian SM , Singer ME , Love TE , Kaiboriboon K . Disparities in access to specialized epilepsy care. Epilepsy Res. 2013;107(1–2):172–180. 10.1016/j.eplepsyres.2013.08.003 24008077 PMC3818489

[70] Nathan CL , Gutierrez C . FACETS of health disparities in epilepsy surgery and gaps that need to be addressed. Neurol Clin Pract. 2018;8(4):340–345. 10.1212/CPJ.0000000000000490 30140586 PMC6105058

[71] Baca CB , Cheng EM , Spencer SS , Vassar S , Vickrey BG , Multicenter Study of Epilepsy Surgery . Racial differences in patient expectations prior to resective epilepsy surgery. Epilepsy Behav. 2009;15(4):452–455. 10.1016/j.yebeh.2009.05.010 19541545 PMC2746976

[72] Shah U , Rathore C , Radhakrishnan K , Baheti N , Kadaba S , Sahu A , et al. A survey of the prevalence and patterns of neuropsychological assessment practices across epilepsy surgery centers in India: toward establishing a national guideline. Epilepsia Open. 2024;9(5):1670–1684. 10.1002/epi4.13005 39012159 PMC11450667

[73] Porrselvi AP , Shankar V . Status of cognitive testing of adults in India. Ann Indian Acad Neurol. 2017;20(4):334–340. 10.4103/aian.AIAN_107_17 29184333 PMC5682734

[74] Dutt A , Evans J , Fernandez AL . Challenges for neuropsychology in the global context. In: Fernandez AL , Evans J , editors. Understanding Cross‐cultural neuropsychology, science, testing and challenges. Current issues in neuropsychology. 2022. Oxford, UK: Routledge, Taylor & Francis Group; 2022. p. 3–18.

[75] Sharma S , Nehra A , Pandey S , Tripathi M , Srivastava A , Padma MV , et al. Neuropsychological rehabilitation for epilepsy in India: looking beyond the basics. Epilepsy Behav. 2024;153:109703. 10.1016/j.yebeh.2024.109703 38452517

[76] Serrano‐Juárez CA , Reyes‐Méndez C , Prieto‐Corona B , Seubert‐Ravelo AN , Moreno‐Villagómez J , Cabañas‐Tinajero JÁ , et al. A systematic review and a Latin American clinical model for teleneuropsychological assessment. Arch Clin Neuropsychol. 2023;38(2):283–300. 10.1093/arclin/acac077 36196778 PMC9619713

