# Supplementary material for: Diagnostic Challenges in the Neuropsychology of Epilepsy: Report of the ILAE Neuropsychology Task Force Diagnostic Methods Commission: 2021–2025
Source: Epileptic Disord. 2025 Jun 13;27(5):729–44. doi: 10.1002/epd2.70052 (PMC12574493; doi:10.1002/epd2.70052)
Supplement: Supplementary file 1 — Appendix S1. [file EPD2-27-729-s002.docx]

Answers:

1. C

2. A

3. C
