# Supplementary material for: Diagnostic Challenges in the Neuropsychology of Epilepsy: Report of the ILAE Neuropsychology Task Force Diagnostic Methods Commission: 2021–2025
Source: Epileptic Disord. 2025 Jun 13;27(5):729–44. doi: 10.1002/epd2.70052 (PMC12574493; doi:10.1002/epd2.70052)
Supplement: Supplementary file 2 — Appendix S2. [file EPD2-27-729-s003.pptx]

## Slide 1
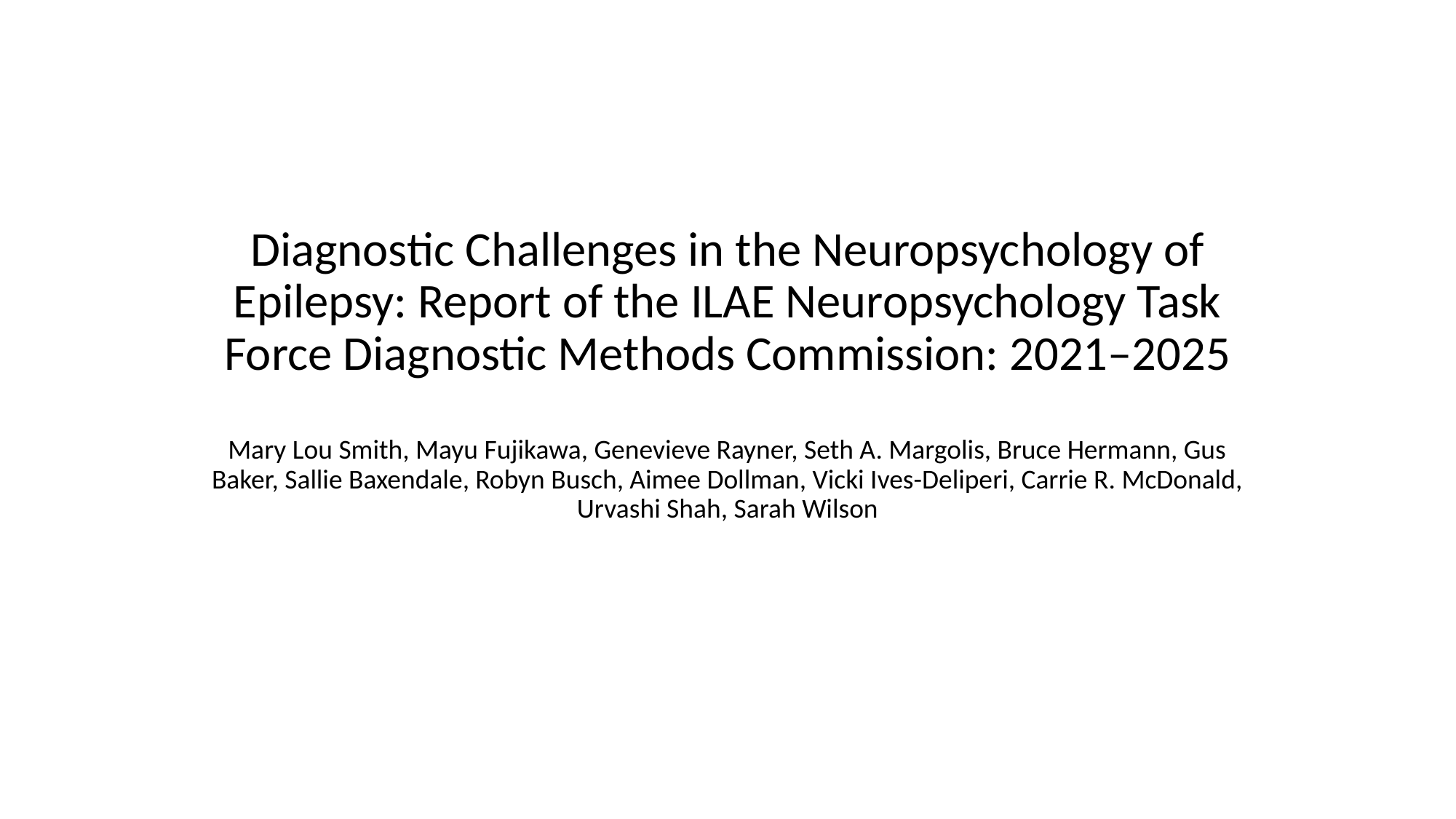

# Diagnostic Challenges in the Neuropsychology of Epilepsy: Report of the ILAE Neuropsychology Task Force Diagnostic Methods Commission: 2021–2025
Mary Lou Smith, Mayu Fujikawa, Genevieve Rayner, Seth A. Margolis, Bruce Hermann, Gus Baker, Sallie Baxendale, Robyn Busch, Aimee Dollman, Vicki Ives-Deliperi, Carrie R. McDonald, Urvashi Shah, Sarah Wilson

## Slide 2
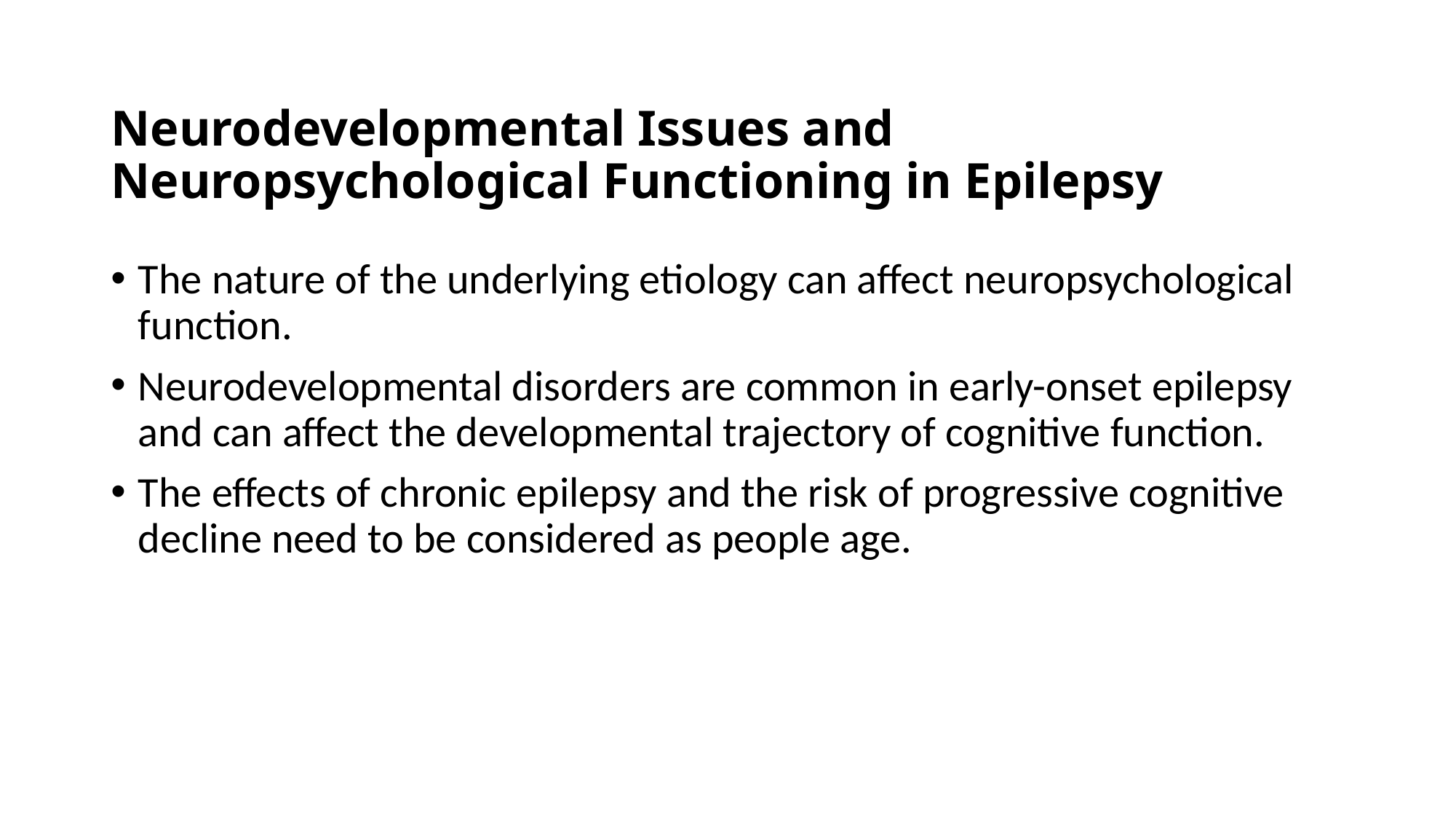

# Neurodevelopmental Issues and Neuropsychological Functioning in Epilepsy
The nature of the underlying etiology can affect neuropsychological function.
Neurodevelopmental disorders are common in early-onset epilepsy and can affect the developmental trajectory of cognitive function.
The effects of chronic epilepsy and the risk of progressive cognitive decline need to be considered as people age.

## Slide 3
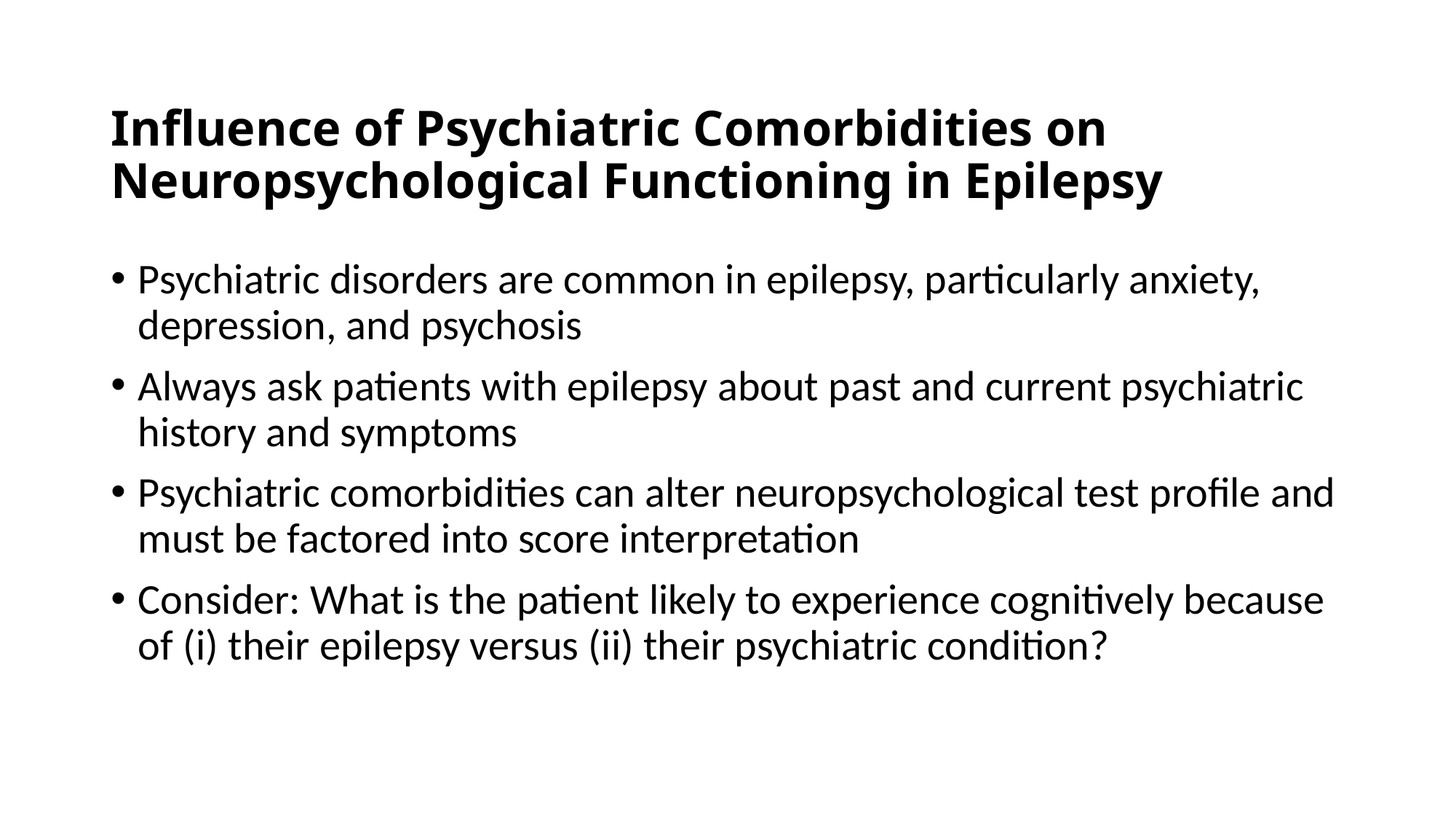

# Influence of Psychiatric Comorbidities on Neuropsychological Functioning in Epilepsy
Psychiatric disorders are common in epilepsy, particularly anxiety, depression, and psychosis
Always ask patients with epilepsy about past and current psychiatric history and symptoms
Psychiatric comorbidities can alter neuropsychological test profile and must be factored into score interpretation
Consider: What is the patient likely to experience cognitively because of (i) their epilepsy versus (ii) their psychiatric condition?

## Slide 4
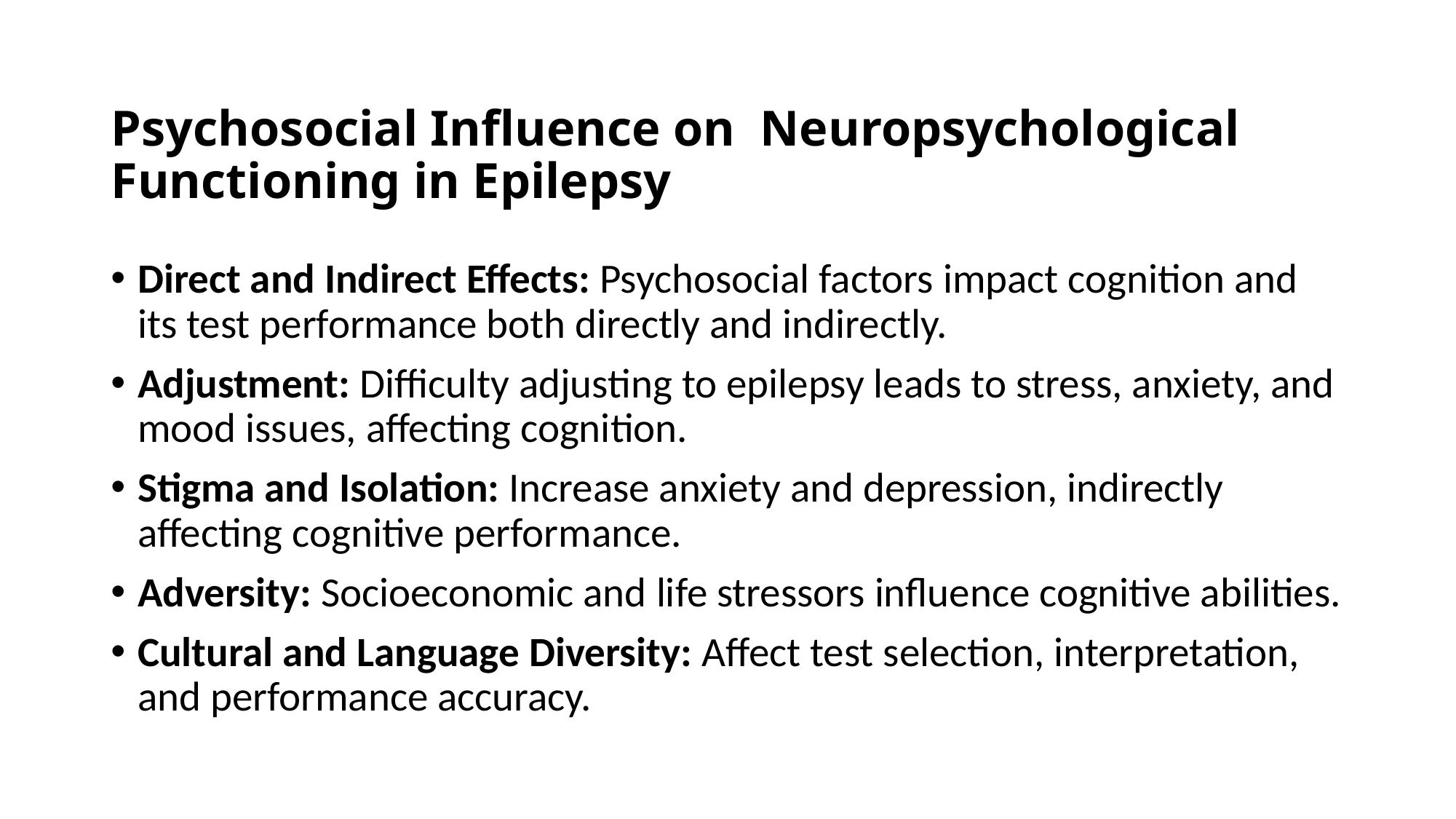

# Psychosocial Influence on Neuropsychological Functioning in Epilepsy
Direct and Indirect Effects: Psychosocial factors impact cognition and its test performance both directly and indirectly.
Adjustment: Difficulty adjusting to epilepsy leads to stress, anxiety, and mood issues, affecting cognition.
Stigma and Isolation: Increase anxiety and depression, indirectly affecting cognitive performance.
Adversity: Socioeconomic and life stressors influence cognitive abilities.
Cultural and Language Diversity: Affect test selection, interpretation, and performance accuracy.
