# Supplementary material for: Diagnostic Challenges in the Neuropsychology of Epilepsy: Report of the ILAE Neuropsychology Task Force Diagnostic Methods Commission: 2021–2025
Source: Epileptic Disord. 2025 Jun 13;27(5):729–44. doi: 10.1002/epd2.70052 (PMC12574493; doi:10.1002/epd2.70052)
Supplement: Supplementary file 3 — Figures S1–S2. [file EPD2-27-729-s001.docx]

Supplementary File: Pre- and post-surgical MRI images

Diagnostic Challenges in the Neuropsychology of Epilepsy: Report of the ILAE Neuropsychology Task Force Diagnostic Methods Commission: 2021–2025

Mary Lou Smith, Mayu Fujikawa, Genevieve Rayner, Seth A. Margolis, Bruce Hermann, Gus Baker, Sallie Baxendale, Robyn Busch, Aimee Dollman, Vicki Ives-Deliperi, Carrie R. McDonald, Urvashi Shah, Sarah Wilson

**Figure 1. Presurgical coronal T2 FLAIR brain MRI showing right mesial temporal sclerosis (*arrow*), characterized by hyperintensity and volume loss in the right hippocampus.**
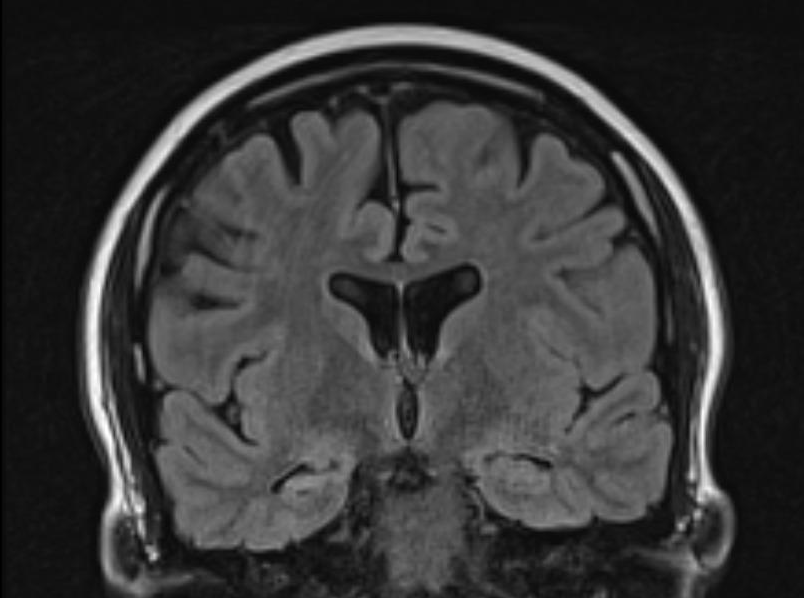


Note: Figure 1 uses a T2-weighted FLAIR sequence, which suppresses cerebrospinal fluid to better highlight parenchymal abnormalities such as sclerosis.

**Figure 2. Post-surgical coronal T2 fat-saturated brain MRI demonstrating laser interstitial thermal ablation (*arrow*) targeting the right amygdala and head of the right hippocampus.**

**
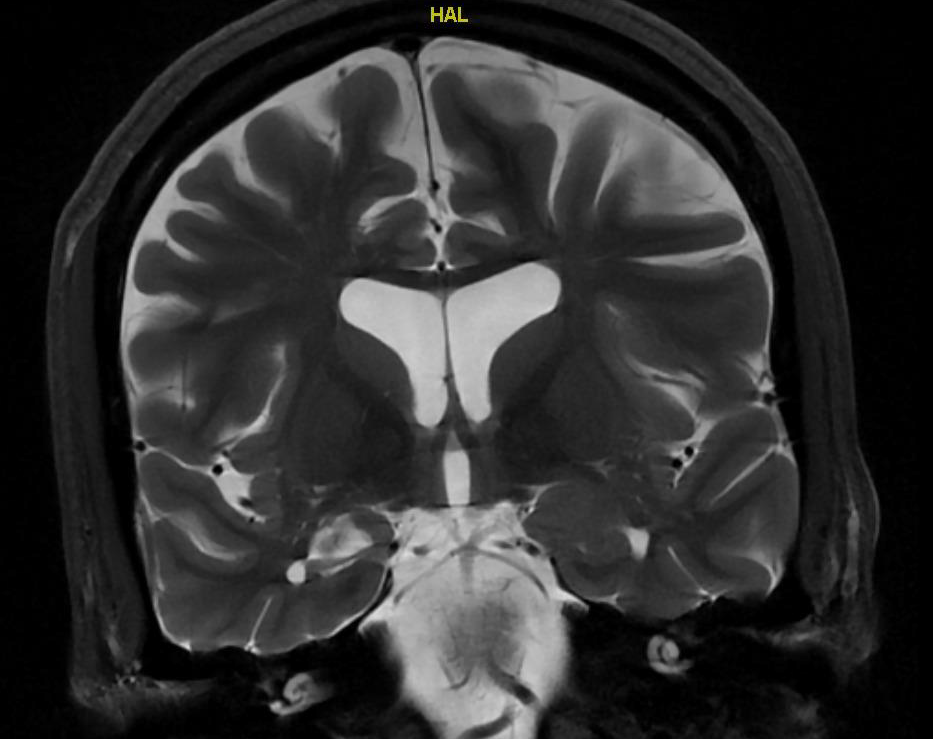
**

Note. Figure 2 uses a T2 fat-saturated sequence to enhance visualization of post-surgical changes and reduce signal from surrounding fat tissue.
